# Evolution of substrate specificity in the Nucleobase-Ascorbate Transporter (NAT) protein family

**DOI:** 10.15698/mic2018.06.636

**Published:** 2018-03-22

**Authors:** Anezia Kourkoulou, Alexandros A. Pittis, George Diallinas

**Affiliations:** 1 Department of Biology, National and Kapodistrian University of Athens, Panepistimioupolis, Athens 15784, Greece.; 2 Department of Botany, University of British Columbia, Vancouver, BC, Canada.

**Keywords:** transporters, L-ascorbate, nucleobase, Aspergillus nidulans, primates

## Abstract

L-ascorbic acid (vitamin C) is an essential metabolite in animals and plants due to its role as an enzyme co-factor and antioxidant activity. In most eukaryotic organisms, L-ascorbate is biosynthesized enzymatically, but in several major groups, including the primate suborder Haplorhini, this ability is lost due to gene truncations in the gene coding for L-gulonolactone oxidase. Specific ascorbate transporters (SVCTs) have been characterized only in mammals and shown to be essential for life. These belong to an extensively studied transporter family, called Nucleobase-Ascorbate Transporters (NAT). The prototypic member of this family, and one of the most extensively studied eukaryotic transporters, is UapA, a uric acid-xanthine/H^+^ symporter in the fungus *Aspergillus nidulans*. Here, we investigate molecular aspects of NAT substrate specificity and address the evolution of ascorbate transporters apparently from ancestral nucleobase transporters. We present a phylogenetic analysis, identifying a distinct NAT clade that includes all known L-ascorbate transporters. This clade includes homologues only from vertebrates, and has no members in non-vertebrate or microbial eukaryotes, plants or prokaryotes. Additionally, we identify within the substrate-binding site of NATs a differentially conserved motif, which we propose is critical for nucleobase versus ascorbate recognition. This conclusion is supported by the amino acid composition of this motif in distinct phylogenetic clades and mutational analysis in the UapA transporter. Together with evidence obtained herein that UapA can recognize with extremely low affinity L-ascorbate, our results support that ascorbate-specific NATs evolved by optimization of a sub-function of ancestral nucleobase transporters.

## INTRODUCTION

Gene duplication and divergence is a major mechanism of evolutionary novelty, providing protein families with an expanded repertoire of activities and novel specificities. The classic view from Susumu Ohno 1,2,3,4 is that duplication creates redundancy, and redundancy provides the material for novelty. Different evolutionary scenarios, such as dosage effects, neo-functionalization or sub-functionalization, might rationalize preservation of gene duplicates 5,6,7,8,9,10. The long-term outcome of neo-functionalization is that one copy retains the original function of the gene, while the second copy, free from selective pressure, explores novel functions. The major criticism of this model is the high likelihood of loss of functionality of the gene product before acquiring a new function, as acquiring functional novelty often needs several intermediate evolutionary steps some of which might introduce deleterious mutations. Novelty might evolve via promiscuous side-activities or sub-functions of proteins, which are optimized in the course of evolution and under specific selective pressures. This model puts emphasis on the multi-functional pre-duplication state of the evolving genes and introduces the possibility that positive selection pressure might drive evolution after gene duplication event. A major issue in the evolution of proteins that remains little answered is how prominent functional changes, which would require the accumulation of multiple serial mutations, take place over the course of time. If many steps in a defined order were necessary for a dramatic functional or specificity change, what would have been the function of ‘intermediate’ proteins? Additional questions remain as to how protein evolution proceeds after gene duplications. Does protein evolution proceed in small steps or in large steps? Is evolution reversible? How does complexity evolve? Is functional specificity determined solely by the substrate or effector binding-site? 7,10,11. One way to approach these questions is to reproduce protein evolution in the laboratory 12. This can be achieved by systematic rational or random mutagenesis and DNA shuffling of specific genes, given a functional assay is designed to follow functional modification, or more efficiently, by direct genetic selection or genetic screens using model microorganisms. What is emerging from these approaches of directed evolution is that functional evolution necessitates conservation of sufficient protein stability, as conformational epistasis might suppress several routes of neo-functionalization 13.

A "horizontal" or phylogenetic approach of molecular and functional comparison of homologous proteins is also a powerful approach to predict protein functions and how these might have evolved. However, this *in silico* approach, by itself, can be misleading as in several protein families, function and mostly specificity cannot be predicted due to overlapping divergent and convergent evolutionary events. Over the last decade, "ancestral protein resurrection" (APR) has developed as a novel strategy to reveal the mechanisms and dynamics of protein evolution 14. APR gives access to a protein’s lost properties that may have transiently arisen over evolutionary time, which in turn can be used to infer the potential selection pressures that resulted in present-day sequences. Thus, APR can identify the causative mutation that resulted in protein neo-functionalization after gene duplication. APR has confirmed that conformational epistasis limits the pathways of functional evolution as neutral mutations may act as ratchets to modification, and additionally supported that evolution seems to proceed from promiscuity to increased specificity, usually via loss-of-function mutations 15.

A still unexplored aspect in protein evolution concerns whether transmembrane proteins, especially those of eukaryotic cells, evolve at similar rates and under similar evolutionary constraints as soluble proteins. This question arises from a number of peculiarities of transmembrane proteins, as for example, their co-translational folding and translocation in the ER membrane, the very complex and highly regulated subcellular traffic, the highly controlled ubiquitination-dependent turnover routes, and their dynamic interaction with specific lipids 16,17,18. These special aspects of transmembrane proteins should in principle impose additional constraints or alternative selective pressures for evolution.

Here, we ask how mammalian L-ascorbate (vitamin C) transporters might have evolved from, apparently, ancestral nucleobase transporters. This question is based on the fact that members of the so-called Nucleobase Ascorbate Transporter (NATs) family 19,20,21,22,23 are highly specific for either nucleobases or L-ascorbate (see also later). This shift in substrate specificity is a dramatic one as these solutes have very different chemical formulas and properties and probably necessitate prominent changes in the architecture of the substrate-binding site and substrate translocation trajectory in the relevant NATs. Our results support a scenario that ascorbate-specific NATs evolved by optimization of a sub-function of ancestral nucleobase transporters.

## RESULTS AND DISCUSSION

### NATs are functionally classified into two distinct specificity groups: nucleobase versus L-ascorbate transporters

More than 24 members of the NAT family have been functionally characterized via direct assessment of their transport activities or indirectly via genetic and physiological studies 19,20,21,22,23 (Table 1). The most extensively studied NATs come from *Escherichia coli* or the filamentous ascomycete *Aspergillus nidulans. *The rest are homologues from other bacteria (*Bacillus subtilis*, *Sinorhizobium meliloti*), ascomycetes (*Aspergillus fumigatus*, *Aspergillus brasiliensis, Candida albicans*) or plants (maize, *Arabidopsis thaliana*). All bacterial NATs are H^+ ^symporters highly specific for either uracil or xanthine or uric acid. The fungal and plant members are H^+ ^symporters specific either xanthine-uric acid, or for adenine-guanine-hypoxanthine-uracil (only in plants) 19,20,21,22,23,24,25. All these NATs exhibit relatively high affinity for their physiological substrates (at the low μM range), but can also recognize with lower affinity several nucleobase analogues. Notably, the relatively specific xanthine-uric acid NATs of fungi can be genetically converted, by single or limited missense mutations, to become broad-specificity promiscuous nucleobase transporters 26,27,28,29. However, no microbial or plant, wild-type or mutant NAT, is capable of transporting L-ascorbate.

**Table 1:**
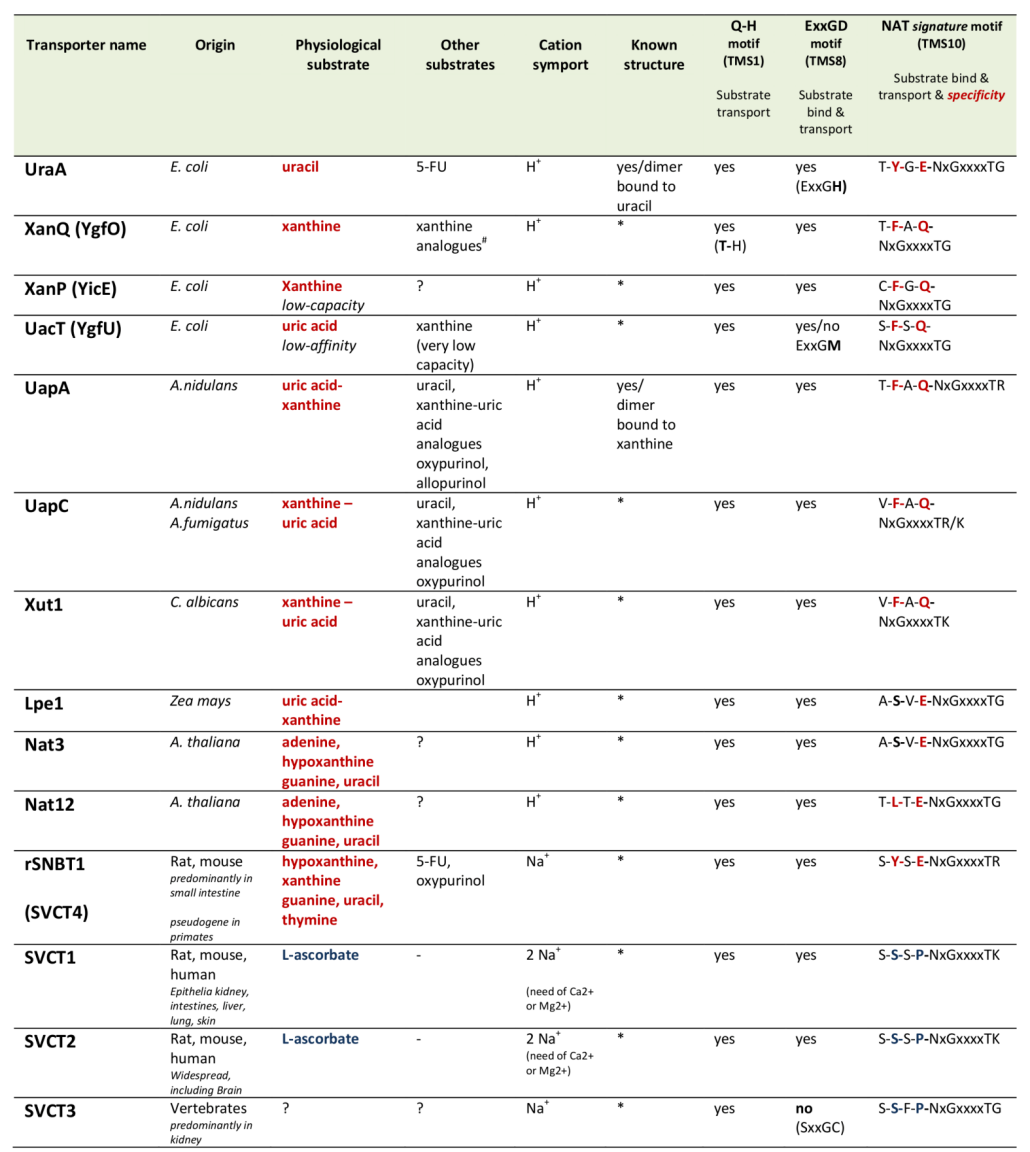
Table 1. Characteristics of biochemically characterized NATs. All NATs shown are high-affinity transporters recognizing their physiological substrate at the low μΜ range, except UacT which is low affinity transporter. NATs marked with an (*) can be modeled, with 100% probability, on the crystal structure of UapA or UraA conforming to the 7+7 inverted repeat fold 30. 5-FU is 5-fluorouracil. Consensus sequences of three motifs involved in function and specificity are shown. Notice that the most significant differences in the NAT *signature motif* of nucleobase *versus *L-ascorbate transporters concern substitutions of the aromatic Phe/Tyr at position 2 and of the polar Gln/Glu with a Pro. ?, not tested. ^#^S. Frillingos pers. com.

In sharp contrast to the microbial and plant proteins, most functionally characterized mammalian NATs, coming from mouse, rat, wild boar or humans, called SVCT1 or SVCT2 (Sodium-dependent Vitamin C Transporters; SLC23 family members), are highly specific for L-ascorbate/Na^+^ 23. However, there is a case reported of a mammalian (rat) NAT transporter, called rSNBT1, which is specific for nucleobases, rather than L-ascorbate 31. Interestingly, the genes encoding orthologues of rSNBT1 in most primates (called *SVCT4*) are truncated pseudogenes. A third distinct homologue of NATs (SVCT3) identified in mammals seems unrelated to either nucleobase or L-ascorbate transport 23. Thus, the emerging view is that all NATs in microorganisms and plants are nucleobase-specific, whereas mammals have both nucleobase and L-ascorbate NATs, but primates have lost via gene truncation the nucleobase-specific ones. However, this picture is little supported by the limited number and biased selection of functionally characterized NATs.

Two members of the NAT family have been crystallized. These are the UraA uracil transporter 32 of *E. coli* and the UapA uric acid-xanthine transporter of *A. nidulans *33. Both proteins form tight dimers, the formation of which is essential for transport activity 33,34,35. The UraA and UapA monomers are made of mostly α-helical segments (TMS) characterized by a 7+7-helix inverted repeat 30. Interestingly, the same topology is also found in transporters with very little primary amino acid sequence similarity with NATs, such as the AzgA-like purine transporters 36 the plant boron transporter Bor1 37, the human Band3 anion exchanger 38, or members of SulP transporter family 39. All these are homodimeric transporters which seem to function via the so-called "elevator mechanism" of transport 40.

### L-ascorbate transporters belong to a phylogenetically distinct NAT clade

We performed an extensive phylogenetic analysis of the NAT family using different datasets and methodologies. NAT sequences used for the analysis are, with very few exceptions, made of 414-650 amino acid residues in length. Selected NAT representatives from all major taxa were also tested, using the HHpred modeling program (https://toolkit.tuebingen.mpg.de), and shown to conform to the 7+7-helix inverted repeat structure of UapA (analysis not shown). Initially we reconstructed a phylogenetic tree using identified NAT homologues in all domains of life, from archaea and bacteria, to fungi, algae, plants and animals (see later). Prominent evolutionary gene-losses of NATs are identified several pathogenic protozoa (e.g. *Trypanosoma, Plasmodium, Leishmania, Giardia*, etc.) and in mesozoa, in several basal fungi including Microsporidia, Cryptomycota and Blastocladiomycota, as well as, in several *Saccharomycetales*, after the whole genome duplication. Interestingly, NAT loss in *Saccharomycetales* is concomitant with gene loss of uric acid oxidase, formation of an allantoin utilization cluster 41 and the "generation" of xanthine dioxygenase (Claudio Scazzocchio, unpublished observations). Other NAT losses in the animal clade are detected in basal Metazoa, such as the Porifera (sponges), some Ctenophora (Eumetazoa), some jaw worms (Gnathostomulida and Xenacoelomorpha), and among early-diverging fish (jawless fish, lampreys or hagfish).

**Figure 1:**
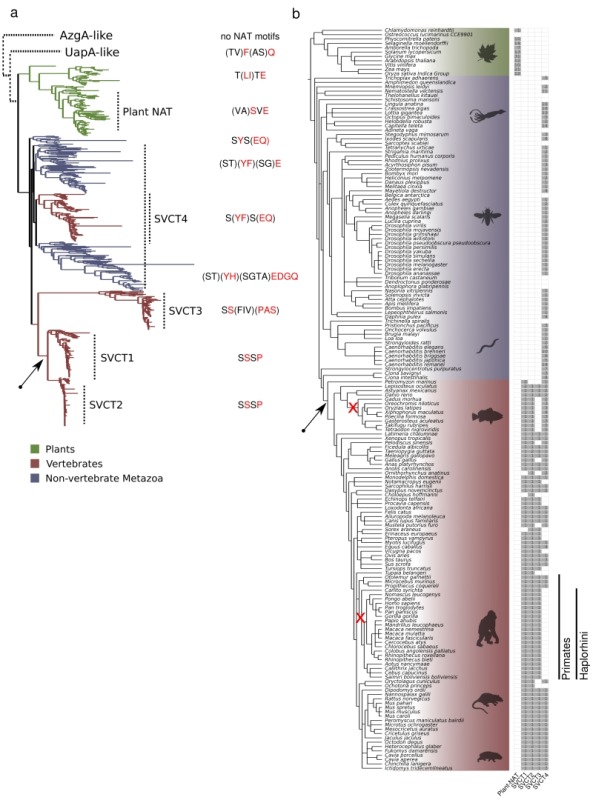
FIGURE 1: Evolution of the SVCT clade in NATs. **(a)** ML phylogenetic tree of the SVCT clade of NATs and the distinct NAT signature motifs. The different SVCT plant and animal NAT subfamilies, as well as a variable consensus of the four first amino acids in the NAT signature motif, are indicated next to the tree. The tree was midpoint rooted, as shown, and the other two clades, UapA-like and AzgA-like, are placed schematically above with dotted lines. The UapA-like group corresponds to canonical microbial NATs and is shown in more detail in Supplementary Figure S1. The AzgA-group is used as an out-group of structurally homologous proteins, which however do not conserve the functional motifs of canonical NATs. For the complete version of the plant and animal NATs see also Supplementary Figure S2. **(b)** Phylogenetic profile of the SVCT clade sub-families for the 171 plant and animal species. The frequency of members in each subfamily is shown next to the NCBI taxonomy tree structure of the 171 species that were searched for NAT domains. The sub-families were defined based on (a) and in reference to the known mammalian transporters (SVCT1-4). Two early losses in two lineages, the SVCT3 in the Teleostean clade Eurypterygii and SVCT4 in the primate suborder Haplorhini, are indicated in the corresponding branches with a red "x". The emergence of the L-ascorbic acid transport specificity is indicated with a black arrow both in the phylogenetic tree (a), as well as in the taxonomy tree (b). The colors (green, red, blue) in both (a) and (b) are as indicated in the figure legend. Both trees were visualized using the ETE toolkit 42.

Figure 1 and Supplementary Figures S1 and S2 highlight our major findings in respect to the distribution of NAT sequences. The first clear observation is that there is a primary evolutionary split between microbial (homologues in archeae, bacteria, algae and fungi, collectively called UapA-like) and plant-animal NATs (Figure 1a). In the microbial/UapA-like group, there are distinct sub-clades for several bacterial groups, archaea, algae and fungi (Supplementary Figure S1). A handful of protists, such as *Dictyostelium discoideum*, are also clustered with specific bacterial clades, suggesting that these might be the products of horizontal gene transfers. A second basic observation is that in the plant-animal clade (SVCT clade), the plants form a clear monophyletic branch including dicots and monocots, as well as, bryophytes (e.g. *Physcomitrella patens*) and some green algae (e.g. *Klebsormidium nitens*) (Figure 1a and Supplementary Figure S2). The animal clade includes several distinct branches. These however can be grouped in four major branches, which herein we named SVCT1, SVCT2, SVCT3 and SVCT4, based on the terminology of the mammalian homologues (Figure 1 and Supplementary Figure S2). The amino acid sequence identity of members of the microbial, plant or animal clades is ~30-92%, whereas the identity between microbial-plant, microbial-animal or plant-animal NATs is ~24-33%.

To gain deeper insights into the evolution of the plant and animal - specific - SVCT clade, we focused on sequences from this clade and deepened our taxon sampling. We used HMMER 43 and the NAT Pfam domain (PF00860) to extract 728 NAT sequences from 171 plant and animal fully sequenced genomes (see methods). After reconstructing a phylogenetic tree using these sequences, we detected and extracted only the 674 sequences comprising the SVCT clade (Figure 1a and Supplementary Figure S2). In the animal clade, an "extended" SVCT4 group (> 45% identity among its members) is widely distributed across Metazoa, and includes members from most vertebrates, non-vertebrates and early diverging animals, as for example, *Trichoplax adhaerens *(the only extant representative of phylum Placozoa, which is a basal group of multicellular metazoa), *Nematostella vectensis *(sea anemone belonging to Cnidaria)*,* *Ciona intestinalis *(sea squirt), *Strongylocentrotus purpuratus *(sea urchin),* Orbicella faveolata *(coral)*, Macrostomum lignano *(flatworm), etc. (Supplementary Figure S2). Most SVCT4-positive species include a single SVCT4 homologue, but there is also significant genus-specific expansion of the group, apparently by gene duplications (e.g. 6-14 paralogues in *Caenorhabditis*, 8 in *Ciona intestinalis*, 5-6 in some fish; Figure 1b). Interestingly, all Haplorhini (anthropoid primates or simians and the tarsier *Carlito syrichta*) have lost functional SVCT4 homologues, due to gene truncations (i.e. generation of pseudogenes; 23). "Patchy" SVCT4 loss is detected also in some non-primate species, but these correspond to genus-specific cases, rather than representing losses in major groups (Figure 1b). Interestingly, SVCT4 loss often coincides with the loss in the ability to synthesize L-ascorbate, although not all known cases that lack this ability lack SVCT4 proteins. Despite the fact that only a single member of the SVCT4 group is functionally characterized (i.e. the rat rSNBT1 nucleobase transporter 31), the conservation of specific functional motifs present in bacterial, plant or fungal nucleobase transporters, allows us to hypothesize that members of the SVCT4 group are specific for nucleobase-related substrates (see later).

The SVCT1 and SVCT2 paralogous subfamilies form a separate clade (~50% identity with SVCT4 in the same species) that includes the well-characterized mammalian L-ascorbate transporters. The SVCT1/SVCT2 group appears exclusively in vertebrates and is probably the result of duplication and diversification of an ancestral animal transporter. Most species maintain one member of SVCT1 and SVCT2 (Figure 1b). Thus, a gene duplication event in the vertebrate ancestor seems to have generated the two sub-families. As the SVCT1 and SVCT2 proteins show high similarity (>68% identity) and all functionally characterized homologues are L-ascorbate transporters, one can speculate that the specificity of L-ascorbate has evolved in the vertebrate ancestor and was retained in fish and tetrapods (see Fig.1). However, whether all extant members of the SVCT1/SVCT2 group are specific L-ascorbate transporters, or whether some SVCT1/SVCT2 homologues transport nucleobases or other substrates, is not formally known.

Finally, SVCT3-like sequences are found in nearly all tetrapod animals and several, but not all, fish (e.g. the *Lepisosteus oculatus, Astyanax mexicanus, Danio rerio, Rhincodon typus; *(Figure 1b). Noticeably, SVCT3 has been lost in some lineages including the Acanthomorpha, a very diverse clade of teleost fishes. The SVCT3 proteins are also the most divergent NAT group in the animal clade, as not only show the lowest overall amino acid sequence similarity with the other clades (< 34% identity with SVCT1/2 and SVCT4), but additionally do not fully conserve the specific amino acid motifs necessary for function and specificity in "canonical" NATs (see later). This might also explain the fact that its phylogenetic position in the tree was rather unstable across different phylogenetic algorithms and parameters (not shown). Thus, SVCT3 members are very probably transporters of substrates other than nucleobases or ascorbate. This is indeed supported by reports showing that the human hSVCT3 does not transport nucleobases or L-ascorbate 23. An analogous "non-canonical" (i.e. lacking the motifs necessary for nucleobase or L-ascorbate transport) divergent NAT group, called UapD-like proteins, has recently been described in fungi 44, while other divergent NAT branches, all of unknown function, seem to exist also in prokaryotes (Stathis Frillingos pers. com. and observations not shown herein).

### The NAT signature motif is differentially conserved in nucleobase and L-ascorbate transporter clades

Previous genetic, molecular, biochemical and structural studies have established that a small number of amino acid residues are essential or critical for NAT function. Most of these fall within three highly conserved motifs in transmembrane segments TMS1, TMS8 and TMS10. The two-amino acid motif **Gln-His** in TMS1 is nearly absolutely conserved in all NATs (only a small number of mostly microbial homologues have a Gln-Gln, Thr-His or Gln-*Φ* versions, where *Φ* is Met or Ile) and seems to be involved in dynamic interactions of TMS1-TMS3-TMS10 necessary for transport catalysis 19,20,21,22,43,44. The TMS8 motif, conforming to the sequence **Glu**-X-X-**Gly**-**Asp**/His/Gly (in bold the most prominently conserved residues), is involved in interactions with substrates and transport 19,20,21,22,46,47. Finally, a longer motif in TMS10, Gln/Glu/Pro-**Asn**-X-**Gly**-X-X-X-X-**Thr**-Gly/Arg/Lys, historically referred as the NAT signature motif 47, is essential for both substrate recognition and transport activity, as well as, substrate specificity 19,20,21,22,46,47,48. The presence of these three motifs is taken, in the present analysis, as an additional criterion for classifying any uncharacterized transporter as a member of the "canonical" NAT family.

The phylogenetic analysis performed herein confirms the presence and exceptional sequence conservation of these motifs in nearly all NAT clades. More specifically, the motifs in TMS1 and TMS8 are highly conserved in all NATs, except from the highly divergent SVCT3 group, which conserves the TMS1 motif, but lacks the TMS8 motif (observations not shown). The motif in TMS10 exists in all NATs but appears in two major versions, especially if we consider three additional upstream amino acid residues (see Figure 1a and Supplementary Figure S2). More specifically, in functionally characterized nucleobase-specific NATs and their close homologues it conforms to the sequence Thr/Ser^1^-Phe/Tyr^2^-Ala/Ser^3^-Gln/Glu/Pro^4^-**Asn**^5^-X-**Gly**^7^-X-X-X-X-**Thr^12^**-Gly/Arg/Lys^13^ (numbers shown are arbitrary). In the L-ascorbate transporter clades (i.e. SVCT1 and SVCT2) this motif is Ser^1^-Ser^2^-Ser^3^-Pro^4^-**Asn**^5^-X-**Gly**^7^-X-X-X-X-**Thr^12^**-Arg^13^. In other words, the most prominent changes, shown underlined, in the two versions of this extended TMS10 motif concern replacements of the aromatic Phe/Tyr^2^ and polar Gln/Glu^4^ (nucleobase transporters) by Ser^2^ and Pro^4^ residues (L-ascorbate transporters). Interestingly, the divergent SVCT3 group has an apparent TMS10 motif more similar to the one found in L-ascorbate transporters rather to that of nucleobase transporters, which also supports its close evolutionary relatedness to them (see Figure 1a).

Based on previous functional 19,20,21,22 and recent structural 32,33,35 studies performed in fungal (e.g. UapA) and bacterial (e.g. XanQ) homologues, Gln/Glu^4 ^is one of the two major NAT residues interacting via a strong bipolar interaction with the nucleobases (the other being the absolutely conserved Glu in the TMS8 motif), while Phe/Tyr^2^ also interacts directly with the nucleobase ring via *pi-pi* stacking. Most importantly, Phe/Tyr^2 ^and Gln/Glu^4^ have proved as key elements in determining the specificity of NATs in respect to different nucleobases. This is best exemplified by specific mutations in UapA, where substitution of Gln^4^ by Glu^4^ (e.g. Q408E) converts this relative specific uric acid-xanthine transporter into a promiscuous transporter capable of recognizing hypoxanthine, guanine or uracil 27, whereas replacement of Phe^2^ by Tyr^2 ^(e.g. F406Y) confers a detectable capacity for hypoxanthine transport 29. Thus, it becomes apparent that more dramatic substitutions of Phe/Tyr^2^ or Gln/Glu^4^, introducing residues as the ones found in L-ascorbate specific NATs (i.e. Ser^2^ and Pro^4^), are expected to result in even more prominent specificity modifications, and might in fact contribute to the “generation” of L-ascorbate transporters. All other conserved residues in the TMS10 signature motif of NATs, including the L-ascorbate transporters, are known to contribute to the kinetic parameters of transport (Thr/Ser^1^, Ala/Ser^3^, Asn^5^, Gly^7^, Thr^12 ^and Gly/Arg/Lys^13^), rather than playing a direct role in determining substrate specificity.

### The differentially conserved residues in the NAT signature motif are essential for function and specificity

No mutation, up to date, in any nucleobase-specific NAT, has led to a transporter capable of recognizing L-ascorbate. However, it should be stressed that there has been no rational genetic design to do so, as most functional mutations were designed or genetically selected to change the specificity of NATs in respect to different nucleobases rather than to L-ascorbate. The only exception is the substitution of Gln^4 ^by Pro^4 ^in the NAT signature motif of the UapA 47 or XanQ transporters 48. In this case, introducing a proline residue led to dramatic reduction (>100-fold) in nucleobase binding and thus no detectable transport activity, while no evidence was obtained for acquisition of L-ascorbate recognition.

**Figure 2:**
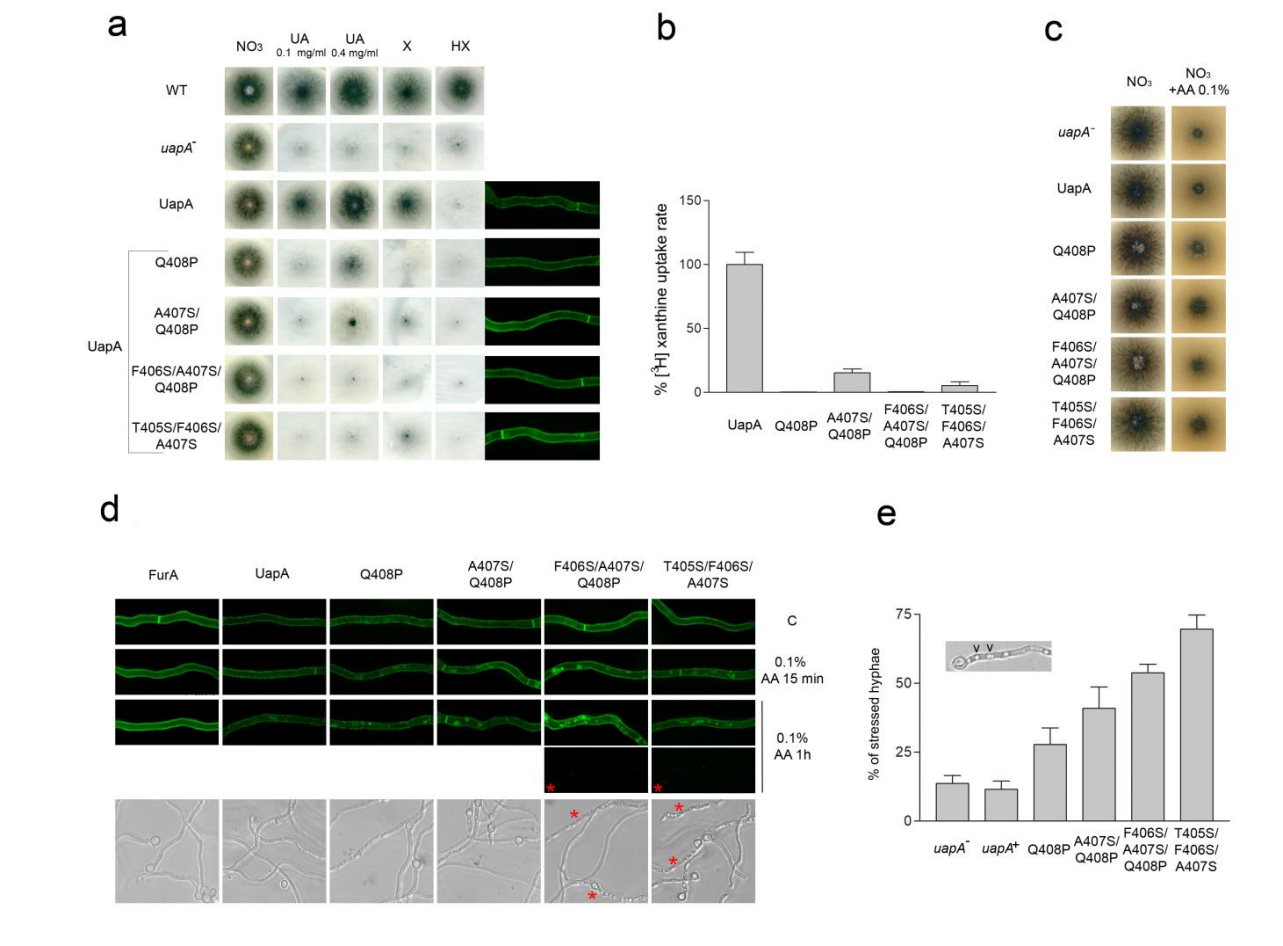
FIGURE 2: Specific residues within the NAT signature motif are critical for UapA function and specificity. **(a) **Growth tests, on standard MM, of isogenic strains expressing wild-type (UapA) or mutant UapA versions (Q408P, A407S/Q408P, T405S/F406S/A407S and F406S/A407S/Q408P), expressed in a genetic background genetically lacking other major purine transporters (i.e. *ΔuapC ΔazgA*). A strain lacking a functional UapA (*uapA^-^*) and a standard wild-type *A. nidulans* strain (WT) are also included in the test as controls. The test depicts growth on 10mM sodium nitrate (NO_3_) or 0.1 mg/ml uric acid (UA), xanthine (X), hypoxanthine (HX), after 48 h at 37°C. A 4-fold higher concentration of UA (0.4 mg/ml) was also used in order to distinguish UapA mutants with highly reduced affinity for substrates (Q408P, A407S/Q408P) from mutants with an apparent total loss of transport activity (T405S/F406S/A407S and F406S/A407S/Q408P). Subcellular localization of wild-type or mutant UapA-GFP versions, as revealed by *in vivo* epifluorescence microscopy, is also shown at the right side of the panel. Notice that in all cases UapA-GFP is stably localized in the plasma membrane and the septa of *A. nidulans* hyphae. For details of sample preparation see Material and methods. **(b)** Comparative UapA-mediated uptake rates of radiolabeled ^3^H-xanthine in strains expressing wild-type (UapA) or mutant versions (Q408P, A407S/Q408P, T405S/F406S/A407S and F406S/A407S/Q408P) of UapA-GFP. Results are averages of three measurements for each concentration point. SD was < 20%. **(c)** Growth tests, at 37°C, on standard MM supplemented with 10 mM sodium nitrate (NO_3_) as nitrogen source in the absence or presence of 0.1% L-ascorbate (AA) of isogenic strains lacking UapA (*uapA^-^*), or expressing wild-type (UapA) or mutant UapA versions (Q408P, A407S/Q408P, T405S/F406S/A407S and F406S/A407S/Q408P). **(d)** *Upper panel*: *In vivo* epifluorescence microscopy following the effect of L-ascorbate (0.1% AA) on UapA-GFP or FurA-GFP subcellular localization. Notice that after 15 min addition of AA clear endocytic turnover of UapA-GFP (i.e. appearance of cytoplasmic structures corresponding to vacuoles; Gournas et al., 2010) is observed in all UapA mutants, but not in wild-type UapA or FurA. Vacuolar turnover is more evident after 1 h of AA in the triple mutants T405S/F406S/A407S and especially F406S/A407S/Q408P, where more and larger vacuoles appear. Notice that after 1 h of AA, moderate vacuolar turnover is also evident in the wild-type UapA, but not in FurA. Noticeably also, 1 h in AA triggers "loss" of GFP fluorescence in a significant fraction (> 50%) of hyphae in in the triple mutants T405S/F406S/A407S and F406S/A407S/Q408P (lowest black boxes marked with a red asterisk). The same effect, albeit significantly reduced, is also seen in A407S/Q408P (36%) and Q408P (22%), while in the wild-type UapA-GFP or FurA-GFP fluorescence loss is < 10% (not shown). *Lower panel*: L-ascorbate (1% AA for 1 h) triggers differential hypervacuolarization and reduction of hyphal width (i.e. cell stress) in UapA mutants and controls. Stressed hyphae are marked with a red asterisk. **(e)** Quantification of the effect of L-ascorbate on hyphal morphology, as recorded for 100 hyphae, of each strain analyzed. In all cases, hypervacuolarization and reduction of the width of hyphae was associated with loss of fluorescence (not shown). The results depicted in the graph confirm the apparent cytotoxicity of L-ascorbate in the triple mutants T405S/F406S/A407S and F406S/A407S/Q408P, where 50-70% of hyphae seem stressed, followed by progressively lower effects on A407S/Q408P (40%), Q408P (27%) and wt UapA (11%).

To further test whether specific variations of the NAT signature motif might be relevant to L-ascorbate recognition, we constructed and/or functionally analyzed a series of relevant UapA mutants. These mutants correspond to the following amino acid replacements in UapA: Q408P 46, A407S/Q408P, T405S/F406S/A407S and F406S/A407S/Q408P. In other words, we modified the first part of the NAT signature motif from TFAQ to TFA**P**, TF**SP**, **SSS**Q or T**SSP** (changes in bold underlined). These mutant versions of UapA were expressed into an appropriate *A. nidulans* strain genetically lacking all endogenous major nucleobase transporters (i.e. UapA, UapC and AzgA) and functionally analyzed in respect to their capacity to transport or bind nucleobases or L-ascorbate, and for proper plasma membrane sorting and stability. Growth tests on purines as sole nitrogen sources can be used as a tool to reveal the functionality of the relative purine transporters. Thus, a control strain expressing a functional UapA (i.e. *uapA^+^*), but no other major purine transporter, grows on uric acid or xanthine, but not on other purines. Figure 2a shows that all UapA mutants tested have lost (T405S/F406S/A407S and F406S/A407S/Q408P) or have dramatically reduced (e.g. Q408P and A407S/Q408P) capacity for growth on uric acid or xanthine. This was confirmed by direct measurements of transport of radiolabeled xanthine (Figure 2b). In addition, none of the mutants tested have acquired any capacity for transport of any other purine, nucleoside or nucleobase-related metabolite (Figure 2a and results not shown). As all UapA mutants tested seem to correspond to abolished or reduced transport function, we wanted to exclude that this not due to misfolding, problematic sorting to the plasma membrane or protein instability. For this, we took advantage of the fact that all mutations analyzed are made in a fully functional UapA protein tagged with GFP. *In vivo* epifluorescence microscopy showed that all mutant versions of UapA analyzed are properly and stably localized in the plasma membrane (right panel in Figure 2a), which in turn confirms that all relevant mutations affect transport function per se.

We additionally tested whether the mutations introduced lead to any growth phenotype that could be related to UapA-dependent L-ascorbate accumulation. Previous results have shown wild-type *A. nidulans* cannot import L-ascorbate, at least when this is externally supplied to concentration up to 1 mM, while higher concentrations of L-ascorbate (5.6-56.8 mM or 0.1-1%) have a progressively moderate inhibitory effect on wild-type *A. nidulans* growth (24, and unpublished observations). Figure 2c shows that none of the UapA mutations analyzed, or the total genetic absence of UapA (*uapA^-^*), alters the growth phenotype of *A. nidulans* on L-ascorbate. We also tried to detect cytoplasmic accumulation of externally supplied L-ascorbate in the mutants or a wild-type strain, using HPLC or a biochemical assay, or by measuring the accumulation of radiolabeled L-ascorbate. In all cases, we did not obtain any evidence of L-ascorbate accumulation (results not shown).

Our results suggested that rational modification of the NAT signature motif is not sufficient, by itself, to confer detectable L-ascorbate accumulation. However, the stable and proper cellular expression of the mutants enabled us to use a sensitive *in vivo* cellular assay, based on substrate-elicited endocytosis, as an indirect measure of the ability of different UapA versions to "recognize", and not necessarily transport at a detectable level, L-ascorbate. This assay is based on the fact that UapA is highly sensitive to endocytic turnover triggered by substrate or ligand binding 49,50. Substrate-elicited transporter turnover is generally promoted by activity-dependent alteration of the conformation of a transporter from an outward-facing to a cytoplasm-facing topology, and can be followed by the appearance of fluorescing vacuoles and early endosome due to sorting of GFP-tagged transporters into these organelles 49,50. Based on this, we tested whether L-ascorbate could promote the endocytosis of wild-type or mutant versions of UapA-GFP, as well as, of a control transporter unrelated to NATs, namely FurA-GFP. FurA is a highly specific allantoin transporter that does not recognize either nucleobase or L-ascorbate 51.

Figure 2d shows that the presences of 0.1% L-ascorbate, for 15 min, promotes significant endocytosis of UapA mutants, but has no similar effect on wild-type UapA or FurA. After prolonged presence of L-ascorbate (1 h), endocytosis of the UapA mutants becomes more prominent, mostly evident in T405S/F406S/A407S and F406S/A407S/Q408P, but also becomes apparent, albeit at a much lower level, in wild-type UapA. Importantly, no endocytic turnover was detected in FurA, in all conditions tested. Additionally, prolonged incubation with L-ascorbate also elicited total loss of fluorescence, associated with a dramatic change in hyphae morphology, in a significant fraction of the UapA mutants. The L-ascorbate-dependent morphological effect, which was insignificant in strains expressing wild-type UapA or FurA, was reflected as a dramatic increase of the number and size of vacuoles and a reduction in the width of hyphae, two changes often seen under severe cell stress. The ascorbate-dependent effect was mostly observed in the triple mutants F406S/A407S/Q408P (in 55% hyphae) and T405S/F406S/A407S (in 68% hyphae) (Figure 2d and 2e), while a similar, albeit reduced, effect is seen in A407S/Q408P (40%) and Q408P (27%) (see Figure 2d and 2e). Given that the most prominent L-ascorbate associated effects are visible in F406S/A407S/Q408P and T405S/F406S/A407S, it seems that the presence of a tandem of Ser residues within the NAT substrate binding site, and the removal of the planar aromatic Phe reside, favor L-ascorbate recognition by the UapA mutants. Importantly, our results show that the L-ascorbate effect on hyphae morphology and the loss of GFP fluorescent are both UapA-specific, as we did not detect the same negative effects in a strain expressing FurA-GFP, which does not express UapA or any other nucleobase transporter (see strain list in Materials and methods).

### UapA can bind L-ascorbate with extremely low affinity: evidence for evolution of L-ascorbate transporters via optimization of a NAT sub-function 

**Figure 3:**
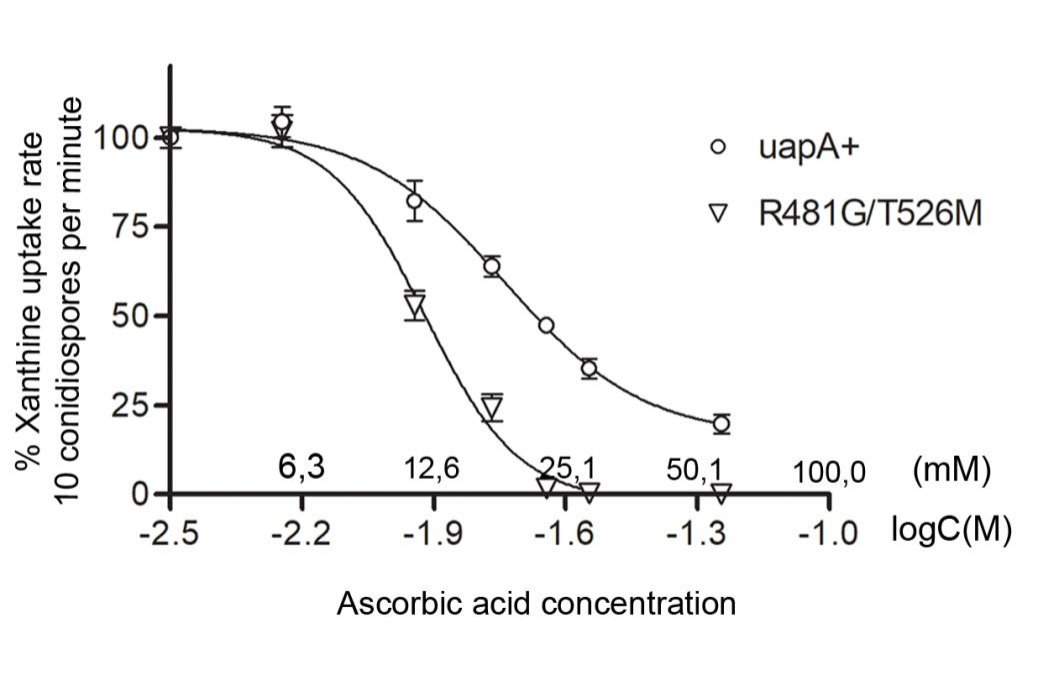
FIGURE 3: L-ascorbate inhibits UapA transport activity. The graph shows dose response curves of [^3^H]-xanthine uptake rate in strains expressing either wild-type UapA or the isogenic mutant UapA-R481G/T526M, in the presence of increasing concentration of non-radiolabeled L-ascorbate (0-56 mM). In both cases, L-ascorbate inhibited UapA transport activity, with *K*_i_ values estimated at 17 and 11 mM for UapA and UapA-R481G/T526M, respectively. Results are averages of three measurements for each concentration point. SD was < 18%. For details of measurements and *K*_i_ estimation see Materials and methods.

The observation that addition of L-ascorbate can also trigger a degree of vacuolarization and endocytic turnover of wild-type UapA-GFP, made us re-examine whether UapA can recognize, even with very low affinity, L-ascorbate. We performed standard competitive inhibition assays to test whether increasing concentrations of L-ascorbate (1-60 mM) inhibit the uptake of radiolabeled ^3^H-xanthine (1 μΜ). Results shown in Figure 3 establish that wild-type UapA can indeed bind L-ascorbate with very low affinity, reflected in a *K*_i_ value of ~17 mM. We repeated the same experiment using a promiscuous UapA mutant capable of recognizing all nucleobases and obtained a moderately increased binding affinity also for L-ascorbate (Ki ~ 11 mM). This mutant (R481G/T526M) has an intact NAT signature motif, but includes two mutations that seem to loosen its specificity by modifying the functioning of putative outward and inward gates 29,33. Thus, UapA can in principle bind L-ascorbate with an affinity approx. 1000-fold lower than the one for its physiological substrates (xanthine or uric acid), or and 10- to 100-lower than its affinity for other nucleobase analogues 47. This finding supports the hypothesis that other nucleobase-specific NATs might also be capable of very low-affinity recognition o L-ascorbate. This in turn supports a model where L-ascorbate NATs evolved via a progressive improvement of a sub-function of nucleobase-specific NATs.

## CONCLUSIONS

Nucleobases are planar molecules with relatively low solubility, whereas L-ascorbate is non-planar and extremely soluble. Such a difference should necessitate prominent changes in the architecture of the substrate-binding site and of the substrate translocation trajectory in the relevant NATs. Our present analysis supports that NAT neo-functionalization took place via progressive improvement of a sub-function (i.e. very low affinity binding of L-ascorbate) in the course of evolution and probably under "new" selective pressures imposed by the evolution of L-ascorbate biosynthesis. This idea leads to many more questions. Are there any present day NATs transporting both substrates equally efficiently? Or are there NATs that can transport L-ascorbate more efficiently than nucleobases? If cases of double specificity exist, could these represent intermediate versions in the evolutionary path from nucleobase to L-ascorbate recognition? Or did only extinct ancestral nucleobase-transporters had this double specificity? NATs that contain transitory variations of NAT signature motif might reflect extant intermediate steps in the evolution of L-ascorbate transporters.

Still many more questions remain to be answered in the direction opened by the present work. Has the specificity shift from nucleobase to L-ascorbate occurred via transporter intermediates that had specificities other than nucleobase or L-ascorbate (could the SVCT3 clade being such a putative case for example?), or even little or no function at all? Can transporters evolve the ability to recognize a new substrate before or after this substrate first appears and how this is related to emergence of substrates via evolution of biosynthetic pathways? Very recently, it was shown that NPF transporters are able to transport both cyanogenic glucosides and glucosinolates, before plants evolved the ability to synthesize glucosinolates 52. Later in evolution, these dual-specificity transporters specialized to transport only glucosinolates. In addition, Jørgensen *et al*. 52 also showed that early glucosinolate transporters could move a broad variety of glucosinolates, but later evolved to only transport particular types. These findings support the idea that evolution of transporter specificity and biosynthetic pathways might evolve together. An analogous case concerning the evolution of specificity in vertebrate corticoid receptors was reported earlier 53,54. Corticoid and glucocorticoid receptors evolved post duplication of a dual specificity aldosterone and cortisol receptor basal to the jawed vertebrate. However, aldosterone biosynthesis did not arise before the advent of tetrapods suggesting that the ancestral receptor evolved affinity towards aldosterone before the hormone was present, possibly as a by-product of the receptors’ affinity towards chemically similar ligands 54.

L-ascorbate biosynthesis seems to have evolved in plants and animals (prokaryotes do not synthesize it) via three major routes. The animal kingdom uses one of these pathways, and photosynthetic eukaryotes (plants, algae and some single-celled organisms) employ the other two. All three pathways proceed via different intermediate molecules, but the two from photosynthetic eukaryotes both use an enzyme called galactolactone dehydrogenase (GLDH), instead of gulonolactone oxidase (GULO), to catalyze the final step 55. Some evidence exists that GULO and GLDH might have evolved from a common ancestor. Interestingly, certain animals, including guinea pigs, most bats, some fish and bird species, as well as humans and many other primates, have lost the ability to synthesize L-ascorbate. These animals obtain ascorbate from their diets 55.

Another important gene loss in vertebrates, possibly relative to NAT evolution, concerns purine catabolism. Most animals oxidize purines into allantoin, which is excreted from their body as the end product of purine catabolism. Humans and other primates are among some groups that lost the gene for uric acid oxidase (i.e. present as a pseudogene), which catalyzes of oxidation uric acid to allantoin 56. This gene loss is partly responsible for the elevated levels of uric acid in blood plasma. Uric acid is a powerful antioxidant and is a scavenger of singlet oxygen and radicals, in a way similar, but not identical, with the action of L-ascorbic acid 57. It has been argued that the loss of uric acid oxidase and elevation of uric acid levels in the body have played an important role in the development of the primate brain and human intelligence due to its protective role against oxidation related to neurotoxic activity. High uric acid levels have also been linked to human life span 57,58. Could it be that evolution of NAT specificity, which shifted from one outstanding antioxidant (uric acid) to another (L-ascorbic acid), and the loss of nucleobase-specific NATs in primates, be related to the co-evolution of relevant metabolic pathways? Relative to such questions, recent evidence supported the co-evolution of the uric acid transporter URAT1 (distinct from NAT transporters) and uric acid oxidase during primate evolution 59. To most of the above questions, a future challenge will be to understand the detailed molecular changes that led to changes in specificity and possibly relate their outcome to the evolution of relative metabolic pathways.

## MATERIALS AND METHODS

### Media, growth conditions and strains

Standard media for *A. nidulans* were used. Media and supplemented auxotrophies were at the concentrations given in the Fungal Genetics Stock Center database (http://www.fgsc.net . Nitrogen sources were used at final concentrations of 10 mM sodium nitrate or 0.5 mM of purines. *A. nidulans* strains used are listed in Supplementary Table S1. The recipient for transformations was the *uapA*Δ *uapC*Δ* azgA*Δ *pabaA1 argB2* mutant strain lacking the endogenous *uapA* gene, as well as the genes encoding a secondary xanthine/uric acid transporter (UapC) and the major purine transporter AzgA. Plasmids carrying *gfp*-tagged wild-type (wt) or mutant versions of *uapA* (i.e. UapA-GFP, 60) were introduced by standard genetic transformation into the recipient strain and transformants were selected on the basis of the *argB* arginine auxotrophy (*argB2*) complementation 61. The strain expressing FurA-GFP, used as a control in Figure 2D, is described in 61. GFP C-terminal tagging has previously shown to have absolutely no effect on UapA or FurA transport kinetics 51.

### Standard nucleic acid manipulations

Genomic DNA extraction from *A. nidulans* was as described in http://www.fgsc.net Plasmid preparation from *E. coli* strains and DNA bands were purified from agarose gels were done with the Nucleospin Plasmid kit and the Nucleospin ExtractII kit according to the manufacturer’s instructions (Macherey-Nagel, Lab Supplies Scientific SA, Hellas). Restriction enzymes were from Takara Bio (Lab Supplies Scientific SA, Hellas). Conventional PCR reactions were done with KAPA-Taq DNA polymerase (Kapa Biosystems, Lab Supplies Sci- entific SA, Hellas). Cloning and amplification of products were done with Kapa HiFi (Kapa Biosystems, Lab Supplies Scientific SA, Hellas).

### Construction and analysis of Aspergillus mutants

All *uapA* mutations appearing in this work were generated by standard site-directed mutagenesis, using specific oligonucleotides (Supplementary Table S2), the QuikChange Mutagenesis Kit and plasmid pAN510-GFP carrying a *gfp*-tagged *uapA* gene, as a template 47,60. The Q408P mutant was constructed previously 27,47. Mutant alleles of *uapA* were introduced by genetic transformation into the recipient strain *uapAD uapCD azgAD pabaA1 argB2*. Selected transformants were purified and those arising from single-copy plasmid integration, as evidenced by PCR and Southern blot analysis, identified 61. Transformants were tested, at 25 or 37°C, pH 6.8, for their ability to grow on MM supplemented with uric acid or other purines as nitrogen sources, or for their general growth phenotype in the presence of L-ascorbate (concentration range from 0.1 to 1 %). Transformants were also tested, using epifluorescence inverted microscopy (using a Zeiss Observer Z1/AxioCam HR R3 camera/Zen lite 2012 software) in respect to UapA-GFP subcellular localization, both in the absence or presence of L-ascorbate (0.01-0.2%) for 15, 60 or 120 min. Samples for epifluorescence microscopy were prepared as previously described, after 16-18 h of growth in standard MM supplements with sodium nitrate as nitrogen source 34.

### Transport assays

UapA mutants were functionally characterized by uptake assays performed in living cells. Radiolabelled [^3^H]-xanthine (22.8 Ci mmol^-1^, Moravek Biochemicals, CA, USA) uptake measurements were performed with *A. nidulans* germinating conidiospores concentrated at 10^7^ conidiospores per 100 ml at 37°C, pH 6.8, at the end of the isotropic growth phase, in which UapA shows maximal expression 62. Competition experiments of [^3^H]-xanthine uptake were carried out with increasing concentrations of unlabeled L-ascrobic acid (1-60 mM). All transport assays were carried out in at least three independent experiments, with three replicates for each concentration or time point and s.d. was < 20 % in all cases.

### Phylogenetic analysis datasets and methods

We selected representative NAT sequences from organisms across all domains of life, representing all major taxa, with particular emphasis in characterized family members of known specificity. For the trees shown in Figure 1 and Supplementary Figure S2, which address in detail the plant and animal NATs (SVCT clades) and the evolution of L-ascorbate transporters, we started by detecting NAT domain containing sequences in complete sequenced genomes, coming from Ensembl release 91, Ensembl Metazoa release 38, and selected species from Ensembl Plants release 38. To detect NAT sequences in the genomes we used HMMER and the PF00860 (*Xan_ur_permease*) Pfam HMM profile, and performed an hmmsearch using the GA (gathering) curated threshold (cut_ga parameter). For the 728 detected sequences, a multiple sequence alignment was built using MAFFT v6.861b in the L-INS-i mode, designed for "set of sequences containing sequences flanking around one alignable domain". The resulting alignment was trimmed using trimAl v1.3 63 using a gap score cut-off of 0.1. The best-fit evolutionary model selection was selected prior to the phylogenetic inference using ProtTest 3 64. Three different evolutionary models were tested (JTT, WAG and LG). The model best fitting the data was determined by comparing the likelihood of all models according to the AIC criterion 65. An ML tree was inferred with RAxML v8.2.11 66 using the best-fitting model. A discrete gamma-distribution model with four rate categories plus invariant positions was used. The gamma parameter and the fraction of invariant positions were estimated from the data. Branch support was computed using an aLRT (approximate likelihood ratio test). We used the exact same methods for the phylogenetic tree of only the SVCT clade, a subset of the previous tree, consisting of 674 sequences, apart from the MAFFT algorithm used, which was the G-INS-I in this case (assumes that entire region can be aligned). ETE toolkit 42 was used for the execution of the phylogenetic pipelines and the visualization of all phylogenetic trees in Figure 1 and Supplementary Figures S1 and S2. ETE was also used for plotting the NCBI taxonomy tree for the 171 species, and the frequency profile of the subfamilies (Fig. 1). For the tree shown in Supplementary Figure S1, which includes all functionally characterized NATs from bacteria and fungi and their closest homologues from other prokaryotic and eukaryotic microorganisms, we obtained the relative sequences by blastP (https://blast.ncbi.nlm.nih.gov) and performed a multiple sequence alignment with MUSCLE v7.0.26 (http://www.megasoftware.net . MEGA was used for testing the aligned sequences for optimal amino acid substitution model. According to the AIC, the LG+G+F model 67 was selected and the tree was created using a maximum likelihood (ML), and visualized by FigTree v1.4.3 (http://tree.bio.ed.ac.uk/software/figtree .

## SUPPLEMENTAL MATERIAL

Click here for supplemental data file.

All supplemental data for this article are also available online at http://microbialcell.com/researcharticles/evolution-of-substrate-specificity-in-the-nucleobase-ascorbate-transporter-nat-protein-family/.
