# Supplementary material for: Evolution of substrate specificity in the Nucleobase-Ascorbate Transporter (NAT) protein family
Source: Microb Cell. 2018 Mar 22;5(6):280–92. doi: 10.15698/mic2018.06.636 (PMC5972032; doi:10.15698/mic2018.06.636)
Supplement: Supplementary file 1 [file mic-05-280-s01.pdf]

Evolution of substrate specificity in the Nucleobase-Ascorbate Transporter (NAT) protein family

Anezia Kourkoulou, Alexandros A. Pittis & George Diallinas

Supplementary Material

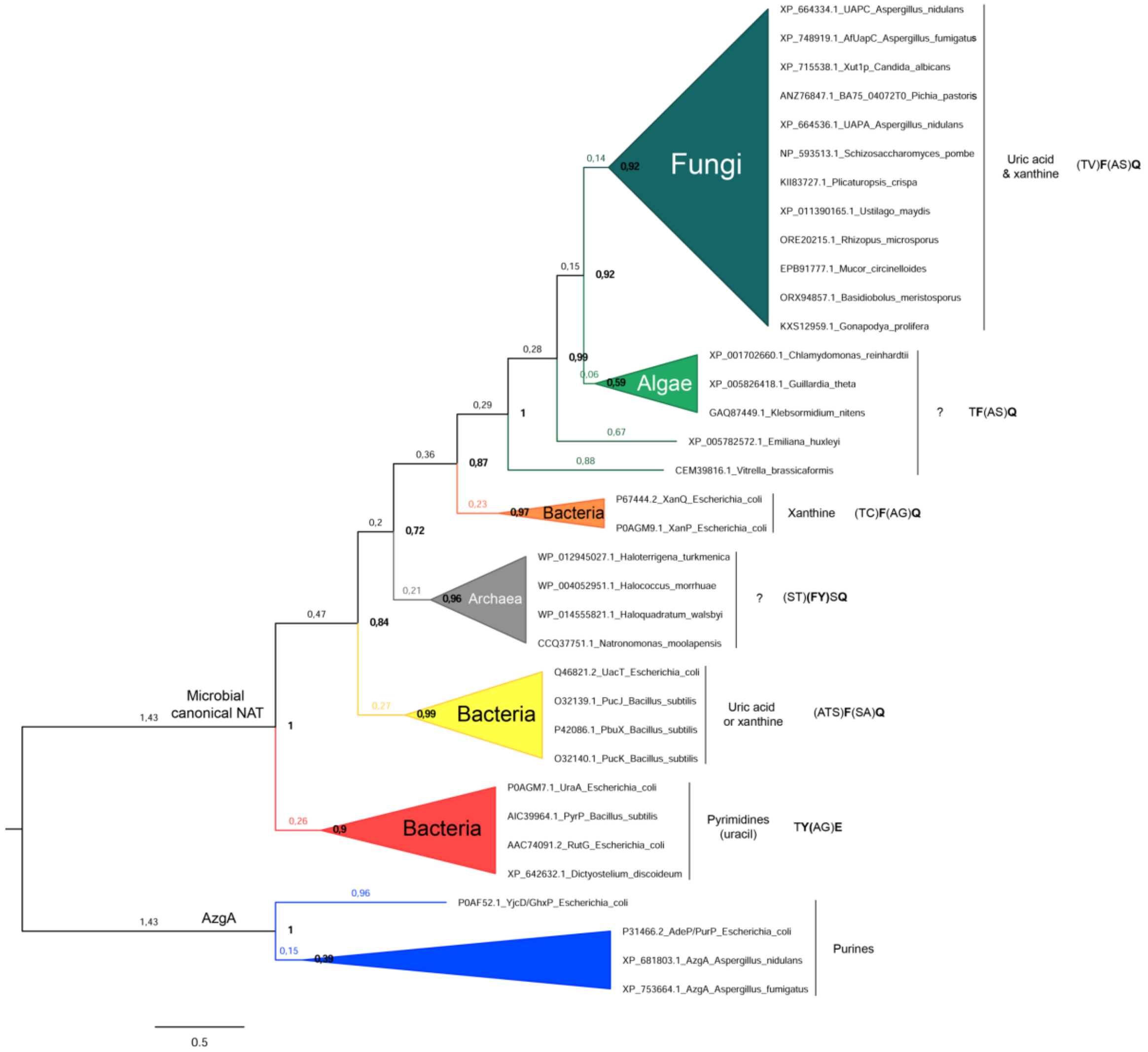

Supplementary Figure S1. Phylogeny of microbial NATs. The tree shown includes all functionally characterized NATs from bacteria and fungi and their closest homologues from other prokaryotic and eukaryotic microorganisms, obtained by blastP (https://blast.ncbi.nlm.nih.gov). The AzgA sequences at the bottom of the tree are functionally characterized purine transporters of the so-called AzgA-like family, which includes proteins structurally similar with NATs, but show very little primary sequence similarity and lack NAT-specific functional motif (closest out-group). Multiple sequence alignments was built from the selected NAT sequences (accession number shown in the figure) using MUSCLE v7.0.26 (http://www.megasoftware.net/). MEGA was also used for testing the aligned sequences for optimal amino acid substitution model. According to the AIC, the LG+G+F model (Le SQ, Gascuel O. Mol Biol Evol. 2008 Jul;25(7):1307-20) was selected and a tree was created using a maximum likelihood (ML), and visualized by FigTree v1.4.3 (http://tree.bio.ed.ac.uk/software/figtree/). Bootstrap values are shown in bold at the nodes of the clades. Evolutionary distances are also shown on the branches of the tree. The variable, four-amino acid, part of the NAT signature motif is shown at the right side of the tree (in bold the residues critical for specificity, see main text). Note that the microbial NAT sequences shown in this tree are collapsed and referred as “UapA-like sequences” in Figure 1 of the main text.

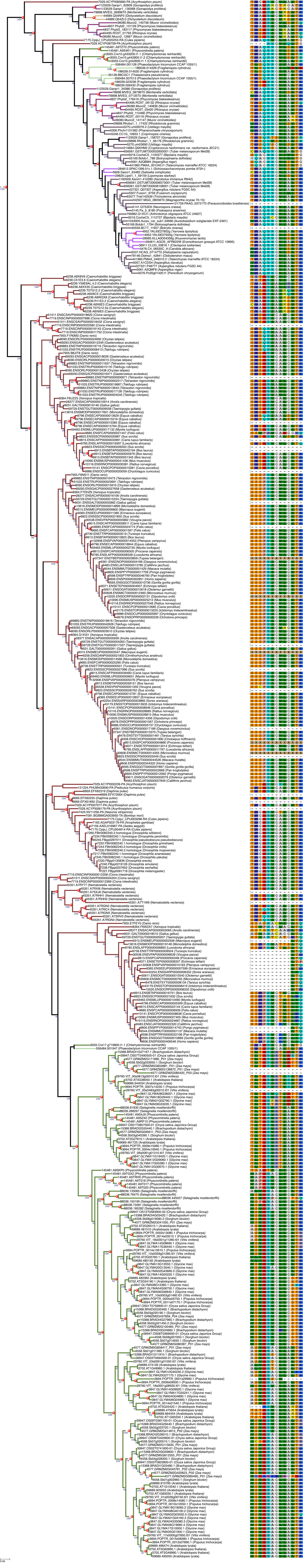

Supplementary Figure S2. Phylogenetic tree of the SVCT clade and the NAT signature motif

The same ML phylogenetic tree as in Figure 1a is shown, here extended and with the sequence - followed by the species - names shown. The protein names correspond to the sequence ids as of Ensembl genomes, or the Uniprot name, for those cases that mapping was successful. Red “spheres” indicate duplication nodes (according to the species overlap algorithm), and the numbers next to the nodes correspond to aLRT support values. On the right, the NAT signature motif is shown for each protein sequence. The ETE toolkit was used for tree visualization [66].

Supplementary Table S1. List of strain used in this study

Strain	Genotype
wt	*pabaA1*
uapA^{-}	*uapAΔ uapCΔ::AfpyrG azgAΔ pabaA1*
UapA	*uapA-GFP-argB uapAΔ uapCΔ::AfpyrG azgAΔ pabaA1*
UapA-Q408P	*uapA-GFP^{-Q408P}-argB uapAΔ uapCΔ::AfpyrG azgAΔ argB2 pabaA1*
UapA-A407S/Q408P	*uapA-GFP$^{-A407S/Q408P}$-argB uapAΔ uapCΔ::AfpyrG azgAΔ argB2 pabaA1*
UapA-F406S/A407S/Q408P	*uapA-GFP$^{-F406S/A407S/Q408P}$-argB uapAΔ uapCΔ::AfpyrG azgAΔ argB2 pabaA1*
UapA-A405S/F406S/A407S	*uapA-GFP$^{-T405S/F406S/A407S}$-argB uapAΔ uapCΔ::AfpyrG azgAΔ argB2 pabaA1*
FurA	*gpdAp-FurA-GFP-panB uapAΔ uapCΔ::pyrG azgAΔ fcyBΔ::argB furDΔ::riboB FurAΔ::riboBΔ cntAΔ::riboB pabaA1*

All strains shown possess the *veA1* mutation which promotes conidiation. *pabaA1* and *argB2* are loss of function mutations leading to auxotrophic requirement of para-aminobenzoic acid and arginine respectively. *AfpyrG* is the *Aspergillus fumigatus* wild type *pyrG* gene(pyrimidine biosynthesis) used as a standard selection marker in *A. nidulans* transformation. *panB* and *riboB* are genes involved in pantothenic acid and riboflavin biosynthesis, used as selection markers for targeted gene knock out. All other genes, encoding transporters, are explained in the text.

Supplementary Table S2. **Oligonucleotides used for the construction of *uapA* mutants.**

	Name	Sequence
1	UapA A407S/Q408P F	5’-CCCCCATGACGACCTTTTCGCCGAACAACGGCGTG-3’
2	UapA A407S/Q408P R	5’-CACGCCGTTGTTCGGCGAAAAGGTCGTCATGGGG-3’
3	UapA F406S/A407S/Q408P F	5’-GACCCCCATGACGACCTCTTCGCCGAACAACGGCGTG-3’
4	UapA F406S/A407S/Q408P R	5’-CACGCCGTTGTTCGGCGAAGAGGTCGTCATGGGGGTC-3’
5	UapA T405S/F406S/A407S F	5’-CAATGACCCCCATGACGTCGTCGTCGCAGAACAACGGCGTG-3’
6	UapA T405S/F406S/A407S R	5’-CACGCCGTTGTTCTGCGACGACGACGTCATGGGGGTCATTG-3’
